# A study on computer vision for facial emotion recognition

**DOI:** 10.1038/s41598-023-35446-4

**Published:** 2023-05-24

**Authors:** Zi-Yu Huang, Chia-Chin Chiang, Jian-Hao Chen, Yi-Chian Chen, Hsin-Lung Chung, Yu-Ping Cai, Hsiu-Chuan Hsu

**Affiliations:** 1 Department of Mechanical Engineering, National Kaohsiung University of Science and Technology, Kaohsiung, Taiwan; 2 Graduate Institute of Applied Physics, National Chengchi University, Taipei, Taiwan; 3 Department of Occupational Safety and Hygiene, Fooyin University, Kaohsiung, Taiwan; 4 Department of Nursing, Hsin Sheng Junior College of Medical Care and Management, Taoyuan, Taiwan; 5 Department of Computer Science, National Chengchi University, Taipei, Taiwan

**Keywords:** Health care, Health occupations, Physics

## Abstract

Artificial intelligence has been successfully applied in various fields, one of which is computer vision. In this study, a deep neural network (DNN) was adopted for Facial emotion recognition (FER). One of the objectives in this study is to identify the critical facial features on which the DNN model focuses for FER. In particular, we utilized a convolutional neural network (CNN), the combination of squeeze-and-excitation network and the residual neural network, for the task of FER. We utilized AffectNet and the Real-World Affective Faces Database (RAF-DB) as the facial expression databases that provide learning samples for the CNN. The feature maps were extracted from the residual blocks for further analysis. Our analysis shows that the features around the nose and mouth are critical facial landmarks for the neural networks. Cross-database validations were conducted between the databases. The network model trained on AffectNet achieved 77.37% accuracy when validated on the RAF-DB, while the network model pretrained on AffectNet and then transfer learned on the RAF-DB results in validation accuracy of 83.37%. The outcomes of this study would improve the understanding of neural networks and assist with improving computer vision accuracy.

## Introduction

In human communications, facial expressions contain critical nonverbal information that can provide additional clues and meanings to verbal communications^1^. Some studies have suggested that 60–80% of communication is nonverbal^2^. This nonverbal information includes facial expressions, eye contact, tones of voice, hand gestures and physical distancing. In particular, facial expression analysis has become a popular research topic^3^. Facial emotional recognition (FER) has been applied in the field of human–computer interaction (HCI) in areas such as autopilot, education, medical treatment, psychological treatment^4^, surveillance and psychological analysis in computer vision^5,6^.

In psychology and computer vision, emotions are classified as categorical or dimensional (valence and arousal) models^7–9^. In the categorical model, Ekman et al*.*^7^ defined basic human emotions as happiness, anger, disgust, fear, sadness, and surprise. In the dimensional model, the emotion is evaluated by continuous numerical scales for determination of valence and arousal. FER is an important task in computer vision that has numerous practical applications and the number of studies on FER has increased in recent years^10–13^, benefiting from the advances provided by deep neural networks. In particular, convolutional neural networks (CNNs) have attained excellent results in terms of extracting features. For example, He et al*.*^14^ proposed the residual neural network (ResNet) architecture in 2015, which added residual learning to a CNN to resolve the issues of vanishing gradient and decreasing accuracy of deep networks.

Several authors have applied neural network models to classify emotions according to categorical models^15–23^ and dimensional models^15,23–26^. Huang^27^ applied a residual block architecture to a VGG CNN to perform emotion recognition and obtained improved accuracy. Mao et al*.*^28^ proposed a new FER model called POSTER V2, which aims to improve the performance of the state-of-the-art technique and reduce the required computational cost by introducing window-based cross attention mechanism and facial landmarks’ multi-scale features. To incorporate more information into the automatic emotion recognition process, some recent studies have fused several modalities, such as the temporal, audio and visual modalities^10,17,18,23,25^, into the algorithm. Moreover, attention mechanisms have been adopted by several studies^17–20,22,25^ for FER tasks. Zhang et al*.*^19^ applied class activation mapping to analyze the attention maps learned by their model. It was found that the model could be regularized by flipping its attention map and randomly erasing part of the input images. Wang et al.^22^ introduced an attention branch to learn a face mask that highlights the discriminative parts for FER. These studies show that attention mechanisms play a critical role in FER. Several approaches for FER utilize self-attention mechanisms to capture both local and global contexts through a set of convolutional layers for feature extraction^29–31^. The extracted features are then used as the inputs of a relation attention module, which utilizes self-attention to capture the relationships between different patches and the context.

However, the practical deployment of facial recognition systems remains a challenging task, as a result of the presence of noise, ambiguous annotations^32^, and complicated scenes in the real-world setting^33–35^. Since attention modules have been proven effective for computer vision tasks, applying attention modules to FER tasks is of great interest. Moreover, in psychology, the facial features for FER by human have been analyzed. The results presented by Beaudry et al*.*^35^ suggest that the mouth is the major landmark when observing a happy emotion and that the eyes are the major landmarks when observing a sad emotion. Similarly, the DNN model extracts discriminative features for FER. It is beneficial to apply class activation mapping to identify the discriminative features learned by the network at each layer. It has been shown that the class activation mapping method can be utilized for localization recognition around the eyes for movement analysis purposes^37,38^. The produced feature maps could provide a better understanding of the performance of the developed model.

In this study, the squeeze-and-excitation module (SENet) was used with ResNet-18 to achieve a relatively light model for FER. This model has fewer trainable parameters (approximately 11.27 million) than the approximately 23 million parameters required for ResNet-50 and the approximately 86 million parameters of the vision transformer. The effectiveness of the proposed approach was evaluated on two FER datasets, namely, AffectNet and the Real-World Affective Faces Database (RAF-DB). Both datasets contain a great quantity of facial emotion data, including those from various cultures and races. The number of images in AffectNet is about 20 times than that of RAF-DB. The images in AffectNet are more diverse and wilder than those in RAF-DB. The neural network was trained to extract emotional information from AffectNet and RAF-DB. A cross-database validation between the AffectNet dataset and the RAF-DB was conducted. The results show that a training accuracy of 79.08% and a validation accuracy of 56.54% were achieved with AffectNet. A training accuracy of 76.51% and a validation accuracy of 65.67% were achieved with RAF-DB. The transfer-learning was applied on RAF-DB with pretrained weight obtained with AffectNet. The prediction accuracy after transfer-learning increases dramatically on the RAF-DB dataset. The results suggest that transfer learning can be conducted for smaller dataset with a particular culture, region, or social setting^36^ for specific applications. Transfer-learning enables the model to learn the facial emotions of a particular population with a smaller database and achieve accurate results. Moreover, the images in AffectNet and RAF-DB with softmax score exceeding 90% were selected to identify the important facial landmarks that were captured by the network. It is found that in the shallow layers, the extracted dominant features are fine lines, whereas in the deep layers, the regions near mouth and nose are more important.

## Database and model

**Figure 1:**
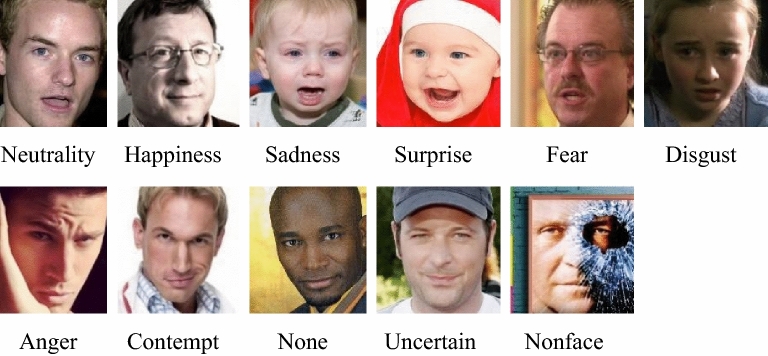
Image categories of the faces contained in the AffectNet database^12^.

The AffectNet database contains 456,349 images of facial emotions obtained from three search engines, Google, Bing and Yahoo, in six different languages. The images were labeled with the following 11 emotions: neutrality, happiness, sadness, surprise, fear, disgust, anger, contempt, none, uncertain, and nonface. Among these emotions, “uncertain” means that the given image cannot be classified into one of the other categories, and “nonface” means that the image contains exaggerated expressions, animations, drawings, or watermarks. Mollahosseini et al*.*^15^ hired annotators to manually classify emotions defined in AffectNet. In addition, AffectNet is heavily imbalanced in terms of the number of images of each emotion category. For example, the number of images representing “happy” is almost 30 times higher than the number of images representing “disgust”. The number of images for each category is shown in Table 1. Figure 1 shows sample images for the 11 emotions contained in AffectNet. In this study, we use seven categories, surprise, fear, disgust, anger, sadness, happiness and neutrality, in AffectNet.

**Table 1:** Number of images in each database^12^.

Category	Number of images in AffectNet	Number of images in RAF-DB
Neutrality	80,276	3,204
Happiness	146,198	5,957
Sadness	29,487	2,460
Surprise	16,288	1,619
Fear	8,191	355
Disgust	5,264	877
Anger	28,130	867
Contempt	5,135	NA
None	35,322	NA
Uncertain	13,163	NA
Nonface	88,895	NA
Total	456,349	15,339

**Figure 2:**
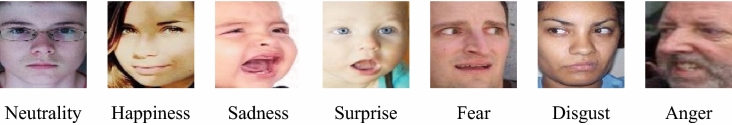
Image categories of the faces contained in the RAF-DB database^37^.

The RAF-DB is provided by the Pattern Recognition and Intelligent System Laboratory (PRIS Lab) of the Beijing University of Posts and Telecommunications^39^. The database consists of more than 300,000 facial images sourced from the internet, which are classified into seven categories: surprise, fear, disgust, anger, sadness, happiness and neutrality. Each of the images contains 5 accurate landmark locations and 37 automatic landmark locations. The RAF-DB also contains a wide variety of information in terms of ages, races, head gestures, light exposure levels and blocking. The training set contains five times as many images as the test set. Figure 2 shows sample images for the seven emotions contained in the RAF-DB. Table 1 shows the number of images used in this article for each emotion from each database.

**Figure 3:**
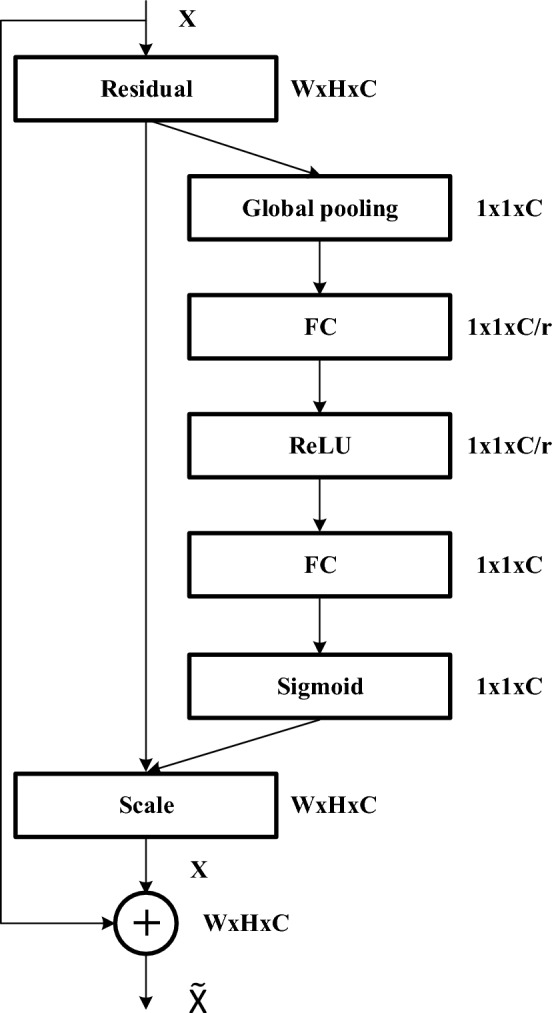
The schema of the SENet inception module.

SENet is a new image recognition architecture developed in 2017^40^. The network reinforces critical features by comparing the correlations among feature channels to achieve increased classification accuracy. Figure 3 shows the SENet architecture, which contains three major operations. The squeeze operation extracts global feature information from the previous convolution layer and conducts global average pooling on the feature map to obtain a feature tensor (Z) of size 1 × 1 × ${\text{C}}$ (number of channels), in which the ${\text{c}} - {\text{th}}$ element is calculated by:

1 $${\text{Z}}_{\text{c}}={\text{ F}}_{{{\text{sq}}}} \left( {{\text{u}}_{{\text{c}}} } \right) \, = \frac{1}{W \times H}\mathop \sum \limits_{i = 1}^{W} \mathop \sum \limits_{j = 1}^{H} u_{c} \left( {i,j} \right)$$

where $F_{sq}$ is the global average pooling operation, $u_{c}$ is the ${\text{c}} - {\text{th}}$ 2-dimensional matrix, W × H represents the dimensions of each channel, and C is the number of channels.

Equation (1) is followed by two fully connected layers. The first layer reduces the number of channels from ${\text{C}}$ to ${\text{C}}/{\text{r}}$ to reduce the required number computations (r is the compression rate), and the second layer increases the number of channels to ${\text{C}}$. The excitation operation is defined as follows:

2 $${\text{S}}_{{\text{c}}} = {\text{ F}}_{{{\text{ex}}}} \left( {{\text{Z}},{\text{ W}}} \right) \, = \sigma (W_{2} \delta \left( {W_{1} Z} \right))$$

where ${\upsigma }$ is the sigmoid activation function, $\delta$ is the rectified linear unit (ReLU) excitation function, and $W_{1}$ and $W_{2}$ are the weights for reducing and increasing the dimensionality, respectively.

The scale operation multiplies the feature tensor by the excitation. This operation captures the significance of each channel via feature learning. The corresponding channel is then multiplied by the gained weight to discern the major and minor information for the computer^38^. The formula for the scale operation, which is used to obtain the final output of the block, is shown as follows.

3 $$\widetilde{{X_{c} }} = {\text{ F}}_{{{\text{scale}}}} \left( {{\text{u}}_{{\text{c}}} ,{\text{ S}}_{{\text{c}}} } \right) \, = {\text{ u}}_{{\text{c}}} \cdot{\text{ S}}_{{\text{c}}}$$

where the dot is the channelwise multiplication operation and $S_{c}$ is the output of the excitation operation.

**Figure 4:**
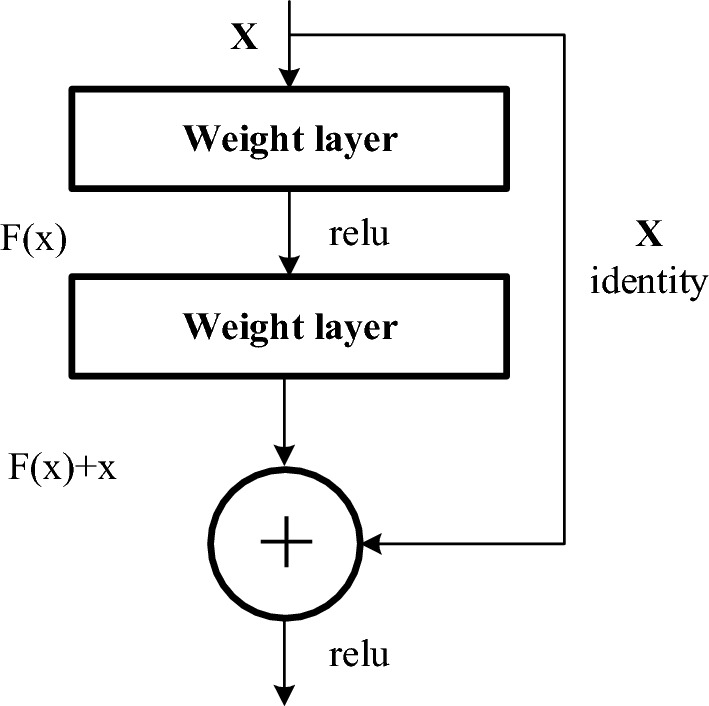
Residual block of the ResNet architecture.

ResNet was proposed by He et al*.*^11^ to solve the vanishing gradient problem in a deep network. ResNet introduces a residual block to a conventional CNN. Figure 4 shows the residual block in the ResNet architecture. The concept of a residual block is to combine the output from the previous convolutional layer with the next convolutional layer in the ResNet. It has been shown in several studies that the residual blocks relieve the vanishing gradient issue encountered by a deeper network. Therefore, the residual blocks have been adopted in several architectures^37,38^.

**Figure 5:**
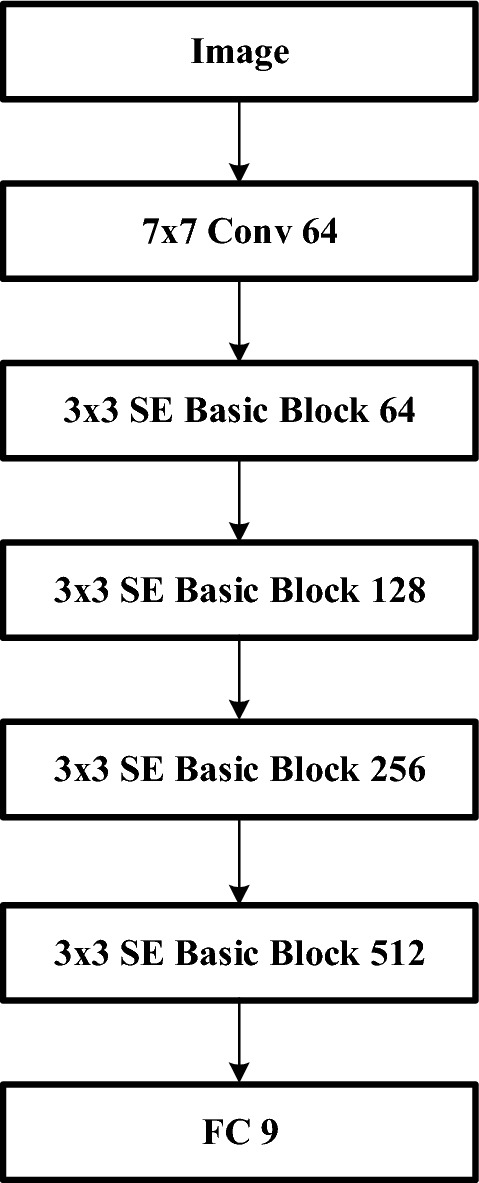
The schema of the SE-Resnet module.

SE-ResNet combines the SENet and ResNet architectures presented above and adds the SE block from SENet to ResNet. The SE block is used to capture the significance of each channel to determine whether it contains major or minor information. The feature information from the previous convolutional layer is then combined with the next layer by the residual block. This method can mitigate the decreasing accuracy caused by the vanishing gradient problem that occurs while increasing the network layers. Figure 5 shows the network architecture of SE-ResNet.

## Experimental method

In this study, we extracted seven categories from AffectNet to ensure that AffectNet and the RAF-DB were validated with identical categories. The SE-ResNet architecture was adopted as the neural network model for training and testing. A comparison and cross-database validation were conducted between RAF-DB and AffectNet. To achieve better performance, the transfer learning technique was used. The model trained on AffectNet was applied as the pretrained model to train RAF-DB.

**Figure 6:**
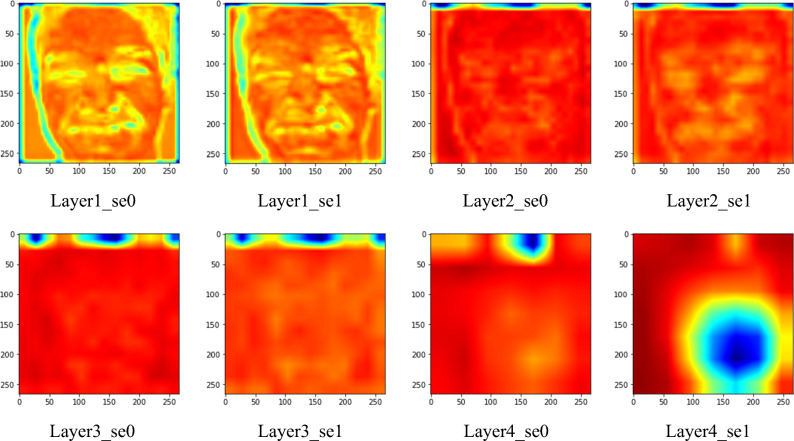
Feature maps of different SE block layers (AffectNet).

**Figure 7:**
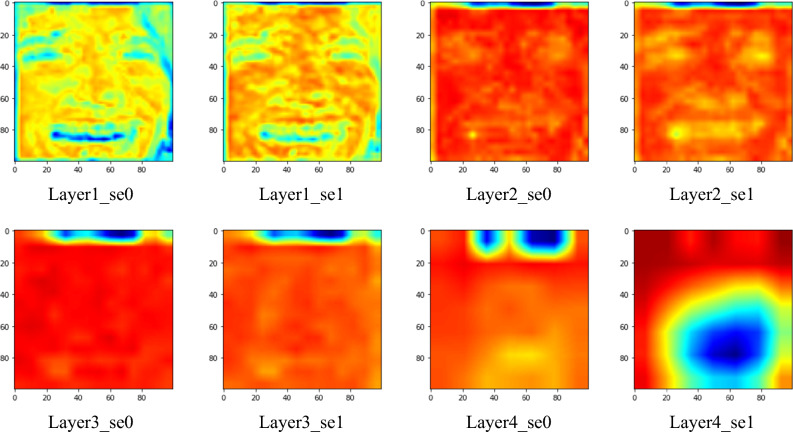
Feature maps of different SE block layers (RAF-DB).

The feature maps derived from each SE block were printed to determine which facial landmarks contain major information for the network. Only facial emotion images with softmax score exceeding 90% were adopted to ensure objectivity and accuracy. Examples of the feature maps printed from AffectNet are shown in Fig. 6. The feature maps printed from the RAF-DB are shown in Fig. 7.

In this experiment, the training hardware was an NVIDIA TITAN RTX 24-GB GPU. The input image size was 256 × 256 pixels with data augmentation. For the training process, the tones of the input images were changed. The images were randomly rotated between + / − 30 degrees, and cropped according to the four corners and the center into five images of the size 224 × 224 pixels. For validation purposes, the input images were cropped from the center to a final size of 224 × 224 pixels. The optimization algorithm and loss function were stochastic gradient descent and the cross entropy loss function, respectively. Twenty epochs were used, and the initial learning rate was set to 0.01. The momentum was 0.9 and the batch size for training was 100.

## Results and discussion

### Cross-database validation

The AffectNet dataset and the RAF-DB were cross-database validated in this study. The model trained on AffectNet was used to predict the RAF-DB, and the model trained on the RAF-DB was used to predict AffectNet. The results are shown in Table 2. Because AffectNet exhibits more diversity in terms of facial emotion data and more images, when the model trained on AffectNet predicted the RAF-DB, an accuracy of 77.37% was achieved, which was significantly higher than the accuracy achieved by directly training on the RAF-DB (65.67%). In contrast, low accuracy (42.6%) was obtained for AffectNet predicted by the model trained on the RAF-DB. The difference can be understood by the fact that the images in AffectNet are more in quantity and more complex.

**Table 2:** Cross-database validation accuracies achieved on AffectNet and the RAF-DB.

Dataset trained on	Dataset tested	Cross-database validation accuracy (%)
AffectNet	RAF-DB	77.37%
RAF-DB	AffectNet	42.6%

### Accuracy

The accuracies achieved on AffectNet and RAF-DB by SE-ResNet were compared in this study. RAF-DB results in a higher accuracy than AffectNet, as shown in Table 3. However, this was expected since the RAF-DB dataset exhibits more constrained images. The accuracy of the proposed model on AffectNet is 56%, which is slightly lower than the 58% accuracy obtained in the original paper^19^ that proposed AffectNet. However, as mentioned in the original paper^15^, the agreement between two human annotators was 60% over 36,000 images. Our result is comparable to this agreement rate.

**Table 3:** Comparison between validation accuracies achieved on AffectNet and the RAF-DB.

Dataset	Validation accuracy (%)
AffectNet	56.54%
RAF-DB	65.67%

Additionally, we performed transfer learning by pretraining the model on AffectNet, followed by training on the RAF-DB. As shown in Table 4, the validation accuracy on the RAF-DB increased by 26.95% ([(accuracy with pretrained model—accuracy without pretrained model)/accuracy without pretrained model = (83.37–65.67) / 65.67] × 100%) and was higher than that of the model trained directly with the RAF-DB. Compared to the accuracy of 76.73% obtained in^21^ by multi-region ensemble CNN, transfer learning with a single network performs better than the ensemble CNN that utilizes global and local features. This result indicates that AffectNet provides useful pretrained weights because of the wide diversity of the dataset. The diverse cultural and racial backgrounds of the images in the AffectNet dataset provides a more representative and inclusive training set, leading to a more robust and accurate recognition system. The result highlights the significance of considering the diversity of data and transfer learning in the development and deployment of FER algorithms.

**Table 4:** Comparison between the validation accuracies achieved on the RAF-DB with/without the pretrained model.

Pretrained model	Validation accuracy (%)
With	83.37%
Without	65.67%

**Figure 8:**
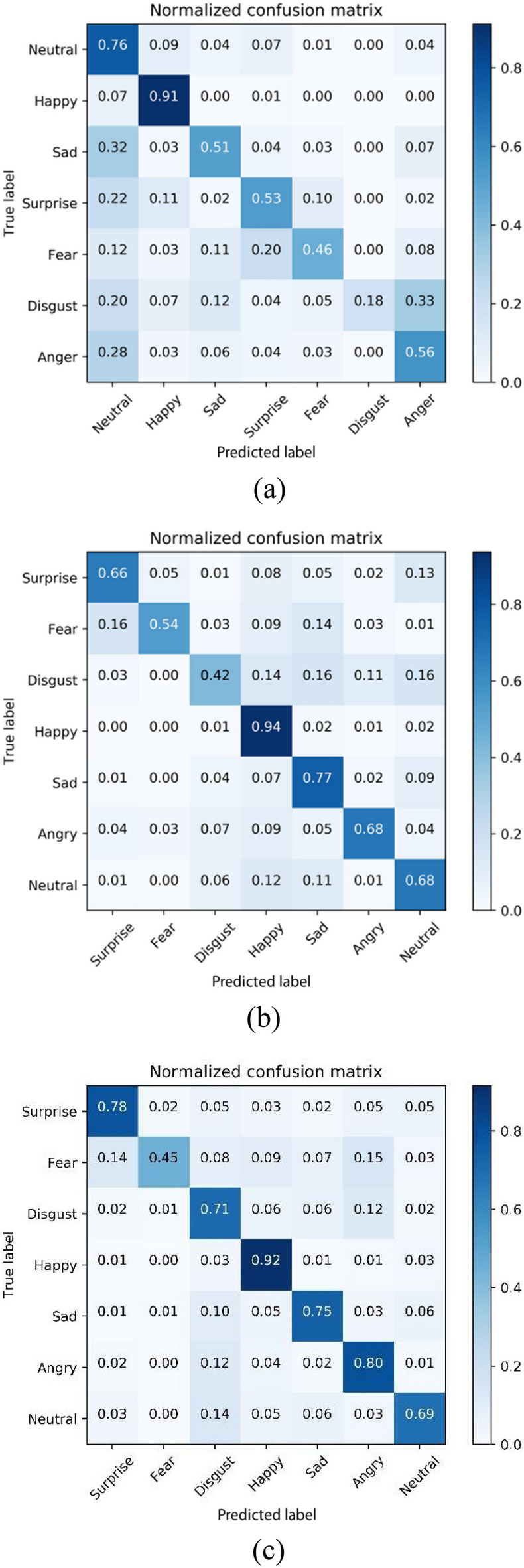
Normalized confusion matrix for AffectNet and RAF-DB (**a**) AffectNet, (**b**) RAF-DB and (**c**) RAF-DB with pretrained model.

The normalized confusion matrices predicted by the model trained on AffectNet for AffectNet and RAF-DB are shown in Fig. 8a and b, respectively. The normalized confusion matrices predicted by the model after transfer learning for RAF-DB is given in Fig. 8c. Figure 8a and b show that the model tends to falsely classify images as “neutral”. It suggests the discriminative features learned from AffectNet are similar between “neutral” and other categories. Moreover, the comparison between Fig. 8b and c shows that after transfer learning, the model classifies the emotions in the RAF-DB in a more accurate and even manner.

It can be seen from the normalized confusion matrices that the classification accuracy is positively correlated with the number of images in the dataset, as given in Table 1. In Fig. 8 a, the AffectNet dataset contains the least number of “disgust” images, which results in the lowest accuracy in the normalized confusion matrix. In contrast, the number of images of the “happy” category is the most in AffectNet and, therefore, yields the highest accuracy in the normalized confusion matrix for this category. The same conclusion can be obtained from Fig. 8b and c for RAF-DB.

### Feature maps

**Figure 9:**
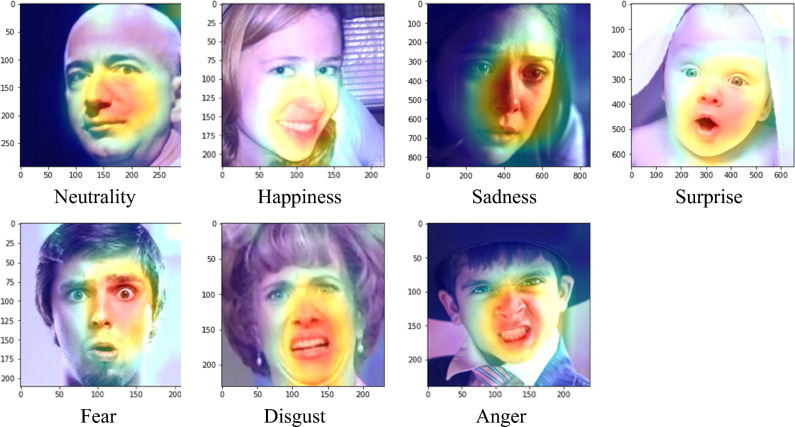
Feature maps with a softmax score greater than 90% (AffectNet).

**Figure 10:**
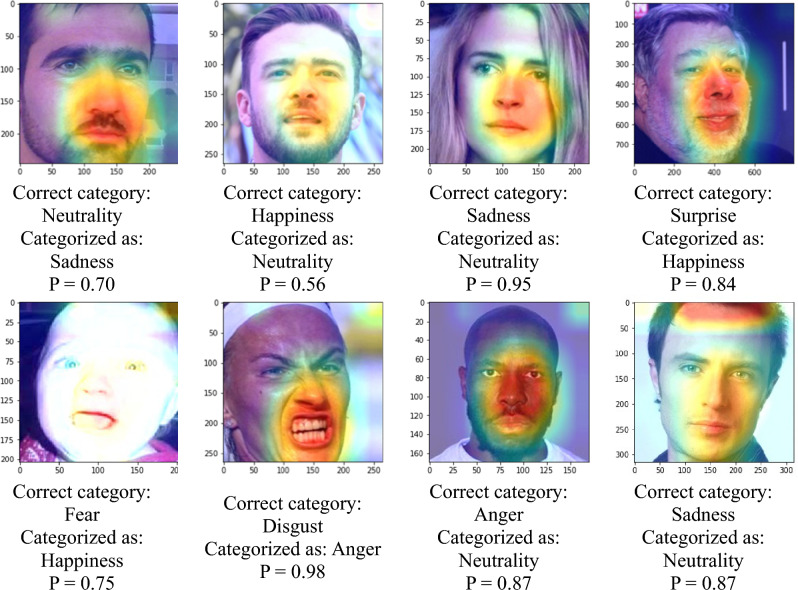
Misclassified feature maps (AffectNet).

This study examines the important features that the network learns to classify facial emotions. The feature maps in AffectNet with softmax scores (P) exceeding 90% are visualized in Fig. 9. It is shown that mouth, nose, and other facial lines are major information, while the eyes and ears for minor information. This is similar to the results found in Beaudry et al*.*^35^ that the mouth is the major landmark when the neural network predicts a happy emotion. The feature maps of misclassified images are also visualized in Fig. 10 for comparisons with those that were correctly classified. By observing the feature maps of misclassified images, it is evident that the important features in the images are similar to those in the correctly classified images. It can be observed from Figs. 9 and 10 that the network tends to detect edges and lines in shallow layers and focuses more on local features, like mouth and nose, in deeper layers.

### Asian facial emotion

**Figure 11:**
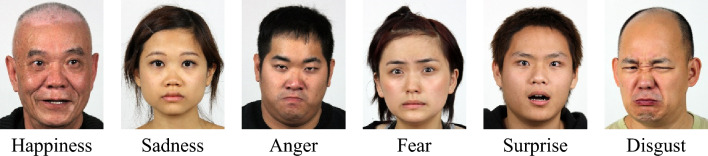
Example images from the Asian facial emotion dataset^39^.

The Asian facial emotion dataset^41^ consists of images of 29 actors aged from 19 to 67 years old. The images were taken from frontal, 3/4 sideways and sideways angles. Figure 11 shows some example images from the Asian facial emotion dataset. The number of images of each class are given in Table 5. There are only six labeled categories in this dataset. The “neutrality” category is not provided in this dataset. Therefore, in the output layer of the model, which was trained to predict the probabilities of 7 categories, the probability for “neutrality” was specified as zero.

**Table 5:** Number of images contained in each category of the Asian descent dataset.

Category	
Happiness	243
Sadness	263
Anger	245
Fear	27
Surprise	220
Disgust	199

**Figure 12:**
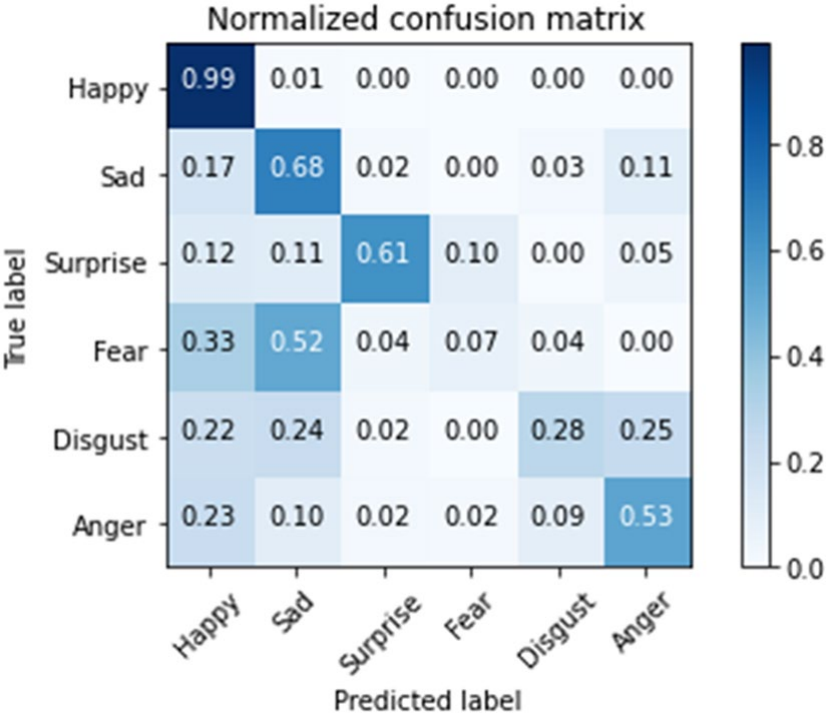
Normalized confusion matrix produced for the Asian facial emotion dataset tested with the model trained on AffectNet.

**Figure 13:**
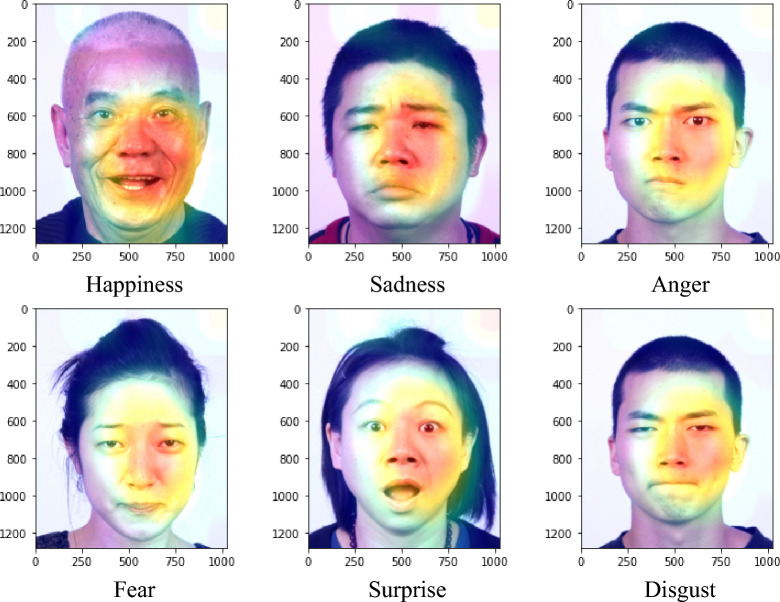
Feature maps produced for the Asian facial emotion dataset.

The Asian facial emotion dataset was tested with the model trained on AffectNet. The images were resized to 256 × 256 pixels and then cropped to 224 × 224 pixels with their faces centered. The derived average accuracy was 61.99%, which was slightly higher than that of AffectNet. Similar to the validation results of AffectNet, the “happy” category yielded the highest score, while “fear” and “disgust” had the lowest scores. The normalized confusion matrix is shown in Fig. 12, and the feature maps are shown in Fig. 13. In contrast with the feature maps of AffectNet, the discriminative locations were not centered around the mouth and nose but were located more on the right half of the face. It shows that the model lacked generalizability for Asian faces in the laboratory setting. This experiment shows that the model trained on AffectNet has limited prediction performance on other datasets.

The process of interpreting facial expressions is also subject to cultural and individual differences that are not considered by the model during the training phase. The feature maps in Figs. 9 and 10 show that the proposed model focused more on the mouth and nose but less on the eyes. To obtain correct FER results, subtle features such as wrinkles and eyes may also be critical. However, the proposed model does not capture features that are far from the mouth or nose. The test results obtained on the Asian face emotion dataset shows that the discriminative regions are skewed toward the right half of the face. This finding indicates that the limited generalizability of the model to Asian faces in the laboratory setting. Although AffectNet is a diverse dataset containing representations from various cultures and races, it is still limited to a tiny portion of the global population. In contrast, the RAF-DB contains similar ethnic groups and settings similar to AffectNet. The validation results obtained on the RAF-DB (77.37%) is better than that on the Asian face emotion dataset. The results show that for datasets with similar ethnic groups, the model trained on a more diverse and wilder dataset (AffectNet) performs better prediction on a more constrained dataset (the RAF-DB in this work).

## Conclusion

This study addresses how the neural network model learns to identify facial emotions. The features displayed on emotion images were derived with a CNN, and these emotional features were visualized to determine the facial landmarks that contains major information. Conclusions drawn based on the findings are listed below.

1. A cross-database validation experiment was conducted for AffectNet and RAF-DB. An accuracy of 77.37% was achieved when the RAF-DB was predicted by the model trained on AffectNet. The accuracy is comparable to the result in^21^. An accuracy of 42.6% was achieved when AffectNet was predicted by the model trained on RAF-DB. These results agree with the fact that AffectNet exhibits more diversity than RAF-DB in terms of facial emotion images. Moreover, transfer learning dramatically increases the accuracy by 26.95% for RAF-DB. The finding highlights the significance of using transfer learning to improve the performance of FER algorithms by training the associated models on AffectNet for pretrained weights.
2. The visualized emotion feature maps show that the mouth and nose contain the major information, while the eyes and ears contain the minor information when the neural network learns to perform FER. This paradigm is similar to how human observes emotions.
3. When comparing the feature maps that were correctly classified (those with softmax scores exceeding 90%) with those that were incorrectly classified, it can be seen that the network model focuses on similar features with no major differences. This result indicates that FER requires the observation of large patches near distinctive areas on a face.

## Data Availability

The datasets applied in this study are available with authorization from the following websites for AffectNet (http://mohammadmahoor.com/affectnet/), the Real-World Affective Faces Database (RAF-DB; http://www.whdeng.cn/raf/model1.html) and the Asian facial emotion dataset (http://mil.psy.ntu.edu.tw/ssnredb/logging.php?action=login). However, restrictions apply to the availability of these data, which were used under license for the current study and thus are not publicly available. The data are, however, available from the authors upon reasonable request and with permission from AffectNet, the RAF-DB and the Asian facial emotion dataset. The training and analysis processes are discussed in the research methodology.
